# PD-1/L1 inhibitor plus chemotherapy in the treatment of sarcomas

**DOI:** 10.3389/fimmu.2022.898255

**Published:** 2022-08-23

**Authors:** Zhichao Tian, Weitao Yao

**Affiliations:** Department of Orthopedics, the Affiliated Cancer Hospital of Zhengzhou University and Henan Cancer Hospital, Zhengzhou, China

**Keywords:** PD-1 inhibitor, PD-L1 inhibitor, chemotherapy, sarcoma, immunochemotherapy

## Abstract

There is an urgent clinical need for new therapeutic regimens for the effective treatment of advanced sarcomas. Accumulating evidence suggests that programmed death receptor-1/programmed death protein ligand-1 (PD-1/L1) inhibitors have synergistic effects with chemotherapy and have been approved for treatment of lung cancer, gastroesophageal cancer, and breast cancer. In this review, we reviewed the synergistic mechanism of PD-1/L1 inhibitors plus chemotherapy in the treatment of cancers, and the application of this combined regimen in several cancers, followed by a summary of the current evidence on the application of this combined regimen in the treatment of sarcomas as well as the main clinical trials currently underway. Based on the findings of this review, we believe that this combined approach will play an important role in the treatment of some subtypes of sarcomas in the future.

## Introduction

Although they account for only approximately 1% of malignant tumors, there are still numerous new sarcoma cases annually (1). More than half of the newly diagnosed sarcoma cases will eventually reach an advanced stage (2). The conventional first-line treatment for advanced sarcomas is chemotherapy. Doxorubicin-based and docetaxel plus gemcitabine regimens are the main chemotherapy regimens for sarcoma treatment (3). However, due to the high heterogeneity of sarcomas (more than 70 subtypes) (4), the overall response rate (ORR) for any chemotherapy regimen is less than 20% (3, 5). The low efficacy of chemotherapy results in a median overall survival (OS) of only approximately 1 year for advanced sarcomas (1, 6). Therefore, there is a strong need for new therapeutic regimens that can effectively treat advanced sarcomas.

Binding of the programmed death receptor‐1 (PD‐1) to its ligand, programmed death protein ligand‐1 (PD‐L1), triggers inhibitory signals that result in reduced proliferation of many anti-tumor immune cells, thereby preventing effector immune cells from killing cancer cells. Blocking this binding can restore effector T-cell activity and anti-tumor immune response, thereby increasing the anti-tumor effects. PD-1/L1 inhibitors are a class of new immune targeting drugs that can effectively block the binding of PD-1 and PD-L1 (7). PD-1/L1 inhibitors have been widely used in the treatment of many malignancies (7). Although PD-1/L1 inhibitors have shown some efficacy in a few sarcoma subtypes, they have limited efficacy in most sarcoma subtypes (8, 9). To improve the efficacy of PD-1/L1 inhibitors in malignant tumors, other treatment methods have been combined with PD-1 inhibitors to achieve synergistic sensitization (10, 11). Among them, the combination of PD-1/L1 inhibitor and chemotherapy has been studied extensively and has been approved by the FDA for the treatment of some cancers such as lung cancer, gastric cancer, esophageal cancer, and breast cancer (12). Although research on the treatment of sarcomas with PD-1/L1 inhibitor plus chemotherapy is still in its infancy, the combination has shown some synergistic effect, which is expected to improve the treatment of advanced sarcomas (13, 14).

In this review, we first summarize the mechanisms underlying the synergistic anti-tumor effects of PD-1/L1 inhibitor plus chemotherapy and its efficacy in various malignant tumors. Then, the evidence for this combination therapy in sarcoma and the ongoing clinical trials are reviewed. Finally, the research strategy and future direction of PD1/L1 inhibitor plus chemotherapy in the treatment of sarcoma are discussed to provide a reference for studies on the treatment of advanced sarcomas.

## Mechanisms of PD-1/L1 inhibitor plus chemotherapy

**Figure 1:**
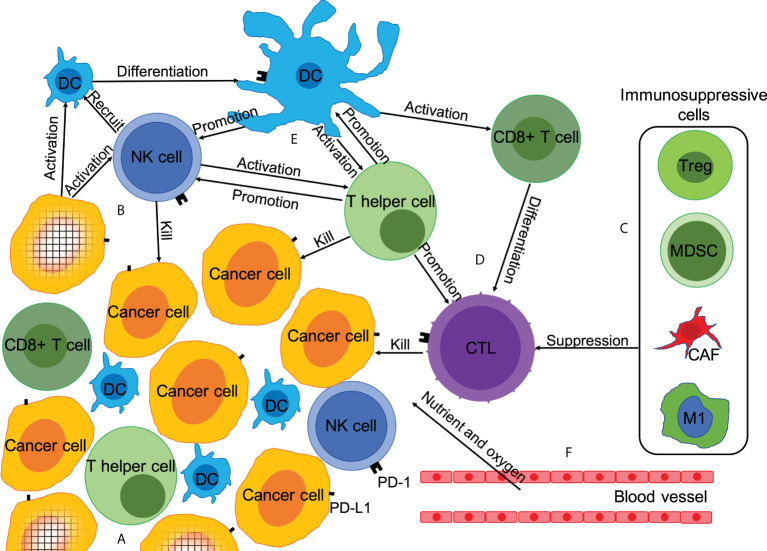
Microenvironment of tumor immunity and mechanism of chemotherapy promoting anti-tumor immune response. Anti-tumor immune response is a complex process, which is affected by the dynamic balance between immune-promoting factors and immunosuppressive factors. Chemotherapy can affect the anti-tumor immune response in at least six ways. **(A)** Reduction in the number of tumor cells; **(B)** Promotion of immunogenic tumor cell death; **(C)** Depletion of immunosuppressive cells; **(D)** Increase in the number and activity of anti-tumor immune effector T cells; **(E)** Enhanced expression of PD-L1 and secretion of anti-tumor immune cytokines; **(F)** Anti-angiogenesis.

Anti-tumor immune response is a complex process, involving cellular and humoral immunity. This anti-tumor reaction generally occurs in the tumor microenvironment (TME) (**Figure 1**). In the TME, tumor cells that generate immune progenitors are first recognized by the natural killer (NK) cells and dendritic cells (DCs) (15, 16). NK cells are activated by recognizing the major histocompatibility complex I (MHC-I) antigen and other tumor antigens expressed on tumor cells. Subsequently, NK cells initiate a cytotoxic reaction to kill the tumor cells directly. Simultaneously, they secrete relevant cytokines as immune helper cells to recruit, stimulate, and regulate DCs, CD4+ T cells, and CD8+ T cells to further activate the anti-tumor immune response (17–19). After DCs are activated by NK cells or tumor antigens, they stimulate CD8+ T cells and induce the anti-tumor immune response of specific cytotoxic T lymphocytes (CTLs). They further regulate the activity and anti-tumor immune response of NK cells and CD4+ T cells *via* various mechanisms (16, 18). After CD4+ T cells are mainly activated by activated DCs, they can directly recognize and kill MHC-II+ tumor cells as well as recruit and activate more NK cells, DCs, and CD8+ T cells, and enhance the tumor cell-killing ability of CTLs (20, 21). Finally, after being activated by DCs, CD8+ T cells are activated into CTLs to kill tumor cells (22, 23). This anti-tumor immune response process is negatively regulated by immunosuppressive cells including myeloid-derived suppressor cells (MDSCs), regulatory T cells (Tregs), and cancer-associated fibroblasts (CAFs) (23, 24). In addition, tumor-associated macrophages (TAMs) and intestinal microbiota can promote or inhibit the tumor immune response (25, 26).

The functions of innate immune cells and adaptive immune cells in the TME can be inhibited by various mechanisms. The most famous one is the PD-1/PD-L1 pathway. As a ligand of PD-1, PD-L1 can be overexpressed on tumor cells, inhibiting the activation and function of CD8+ T cells to avoid the anti-tumor immune response (27). PD-1/L1 inhibitors are one of the most promising and attractive anticancer immune checkpoint blockers (7). There is increasing evidence that apart from T cells, PD-1/L1 inhibitors can also directly or indirectly promote the function of other immune cells, thereby inhibiting tumor progression **(** **Figure 1**) (28).

Chemotherapeutic drugs have extensive effects on the TME. Numerous studies have confirmed the synergistic mechanism between chemotherapy and anti-tumor immunotherapy (12). We have summarized and listed these mechanisms below (**Figure 1**), based on the research results of various chemotherapeutic drugs in different malignant tumors. However, it is impossible for any one type of chemotherapeutic drug to have a synergistic mechanism with PD-1 inhibitor that can cover all the aspects listed below. Some chemotherapeutic drugs can even inhibit the effect of PD-1 inhibitors under certain conditions. Since the anti-tumor immune response is highly complex, it is impossible for any mechanism to exist alone. The mechanisms listed below are widely related, and some mechanisms may even be mutually causal.

## Reduction in the number of tumor cells

Tumor cells can compete with immune cells for nutrients and key metabolites in the TME, resulting in impaired metabolism, limited proliferation, and weakened anti-tumor immune function of immune cells (29, 30). The reduction in the tumor tissue volume and number of tumor cells caused by chemotherapy can not only reduce the expansion of the immune cells and anti-tumor immune pressure but also reduce the chances of tumor cell variation and immune escape. Additionally, larger tumor lesions can inhibit the function of the systemic immune system, and reduction in the tumor lesions caused by chemotherapy can reduce or relieve this inhibition (31).

## Promotion of immunogenic tumor cell death

Chemotherapeutic drugs mainly cause apoptosis of tumor cells. Tumor cells injured by chemotherapy first transmit danger signals to antigen-presenting cells in the form of endogenous damp-associated molecular patterns (DAMPs). DAMPs represent changes in the surface and microenvironment of the cells in response to stress, including that induced by chemotherapy, which is reflected by the expression of new (usually unexpressed) membrane-bound or secreted proteins, such as type I interferon. Antigen-presenting cells (mainly DCs) recognize these changes on the surface and microenvironment of injured tumor cells and activate tumor-specific immune responses (32). The dead tumor cells also release a large number of cytoplasmic and nuclear proteins, cancer cell cytosol, and nucleic acids (including DNA and different forms of RNA), which promote the cross presentation of DC-mediated tumor antigens to CTLs, ultimately promoting the recognition and clearance of residual cancer cells (33).

In conclusion, chemotherapy leads to a stress response and apoptosis, resulting in many new tumor immune antigens on the cell surface and TME. These antigens stimulate an anti-tumor immune response, which can co-operate with the anti-tumor immune response activated by PD-1/L1 inhibitors to kill the residual cancer cells (12).

## Depletion of immunosuppressive cells

In the normal human body, there is always a dynamic balance between the activation and inhibition of the immune system. The main immunosuppressive cells associated with the anti-tumor immune response include MDSCs, Treg cells, CAFs, and TAMs (23, 28). Chemotherapy can significantly reduce these cells in the peripheral blood and TME, and reduce the inhibitory factors of the anti-tumor immune response (34, 35). Nevertheless, chemotherapy can lead to the loss and reduction of almost all immune cells (35). If the timing is correct, PD-1/L1 inhibitor can first activate the function of the effector T cells and promote their quantitative expansion, thereby ensuring a more efficient anti-tumor response in the TME lacking immunosuppressive cells (32, 34).

## Increased number and activity of anti-tumor immune effector T cells

The various immune cells in the body are in dynamic balance. Reduction in the number of other immune cells can make more space for effector T cells and expose effector T cells to more immune- and growth-promoting cytokines (36). Several studies have confirmed the above hypothesis (37, 38). Furthermore, CD8+ T cells demonstrate relative elasticity to the effects of many cytotoxic chemotherapeutic drugs. Although these drugs may cause a temporary decrease in the number of CD8+ T cells in the blood, in most cases, the CD8+ T cell population recovers well, mainly due to the expansion of the effector memory pool. The chemotherapeutic agents can even induce enhanced activation of the CD8+ T cells (39, 40). Consequently, after a recovery period, the CD8+ T cell pool may actually be enhanced after some chemotherapy regimens, which is due to an increase in the cytotoxic activity and transfer of the cell population from terminal effector cells to effector memory cells (35). If PD-1/L1 inhibitor is administered at this time, the higher proportion of effector T cells are more effective in responding to the effect of the PD-1/L1 inhibitor. However, different doses of chemotherapeutic drugs have significantly different effects on effector T cells. Generally, the higher the dose of the chemotherapeutic drug, the more serious the damage to effector T cells (38). Therefore, when chemotherapy is combined with a PD-1/L1 inhibitor, the dose of chemotherapy drugs should not be too high.

## Enhanced expression of PD-L1 and secretion of anti-tumor immune cytokines

Many studies have confirmed that chemotherapeutic drugs can enhance the expression of PD-L1 in tumor tissues (41, 42). A high expression of PD-L1 has been considered an effective marker for PD-1/L1 inhibitor use in various malignant tumors (43, 44). Chemotherapy can also lead to changes in the various cytokine lineages in the TME (41). These alterations are complex and even contradictory, promoting or inhibiting anti-tumor immunity. For example, in some studies, cisplatin has been shown to enhance the release of various chemokines that attract T cells and enhance anti-tumor immunity (38, 45). In other studies, it led to a decrease in tumor necrosis factor α levels or an increase in the pro-inflammatory cytokine levels that promote tumor proliferation (46, 47). Obviously, these changes are related to the different doses of the different chemotherapeutic drugs.

In conclusion, under the influence of appropriate doses of appropriate chemotherapeutic drugs, PD-L1 expression and cytokine lineage in the TME can change in the direction of promoting the efficacy of PD-1/L1 inhibitors.

## Anti-angiogenesis

Anti-angiogenesis can lead to tumor vascular degeneration, tumor necrosis, and improve antigen presentation by DC cells (48). The combination of antiangiogenic agent and PD-1 inhibitor also enhanced the presence and activation of CTLs in TME, further enhancing the antitumor effect (49, 50). Existing research has shown that chemotherapeutic drugs can inhibit tumor angiogenesis, even at low doses (51, 52). This can not only make it easier for T lymphocytes to penetrate the vascular endothelial barrier to reach the tumor lesions but also lead to hypoxia and nutrient deprivation of the tumor tissue and TME (53). This is a double-edged sword for tumor immunity. Hypoxia may increase the activity of CTLs and may also lead to a TME-promoting tumor proliferation (54). At present, only a few studies have confirmed the synergistic effect of chemotherapeutic drugs and PD-1 inhibitors in this regard (55). The mechanism of synergy in this area needs more in-depth research.

## Efficacy of PD-1/L1 inhibitor plus chemotherapy in different cancers

Although the potential synergistic mechanisms of PD-1/L1 inhibitor plus chemotherapy are numerous, they must achieve better outcomes in clinical practice to be meaningful. At present, PD-1/L1 inhibitor plus chemotherapy has completed phase 3 clinical trials in many cancers (**Table 1**).

**Table 1:** Partial phase 3 clinical trials of PD-1/L1 inhibitor plus chemotherapy in different cancers.

Type of cancer	Name of PD-1/L1 inhibitor	Names of chemotherapy drugs	Clinical outcomes	References
Squamous NSCLC	Pembrolizumab	Carboplatin plus paclitaxel or nab-paclitaxel	Median PFS was 6.4 months in the pembrolizumab-combination group and 4.8 months in the placebo-combination group.	(56)
Non-squamous NSCLC	Pembrolizumab	Pemetrexed and a platinum	Median PFS was 8.8 months in the pembrolizumab-combination group and 4.9 months in the placebo-combination group.	(57)
SCLC	Atezolizumab	Carboplatin and etoposide	Median PFS was 5.2 months in the atezolizumab-combination group and 4.3 months in the placebo-combination group.	(58)
SCLC	Durvalumab	Platinum and etoposide	Median OS was 13.0 months in the durvalumab plus platinum-etoposide group versus 10.3 months in the platinum-etoposide group.	(59)
Gastric, gastroesophageal junction, or esophageal adenocarcinoma	Nivolumab	Oxaliplatin and capecitabine or fluorouracil	Nivolumab plus chemotherapy resulted in significant improvements versus chemotherapy alone in OS (13.1 vs 11.1 months) in patients with a PD-L1 CPS of 5 or more.	(60)
Esophageal squamous cell carcinoma	Pembrolizumab	5-fluorouracil and cisplatin	Pembrolizumab plus chemotherapy was superior to placebo plus chemotherapy for OS in all randomized patients (12.4 months vs 9.8 months).	(61)
Triple-negative breast cancer	Pembrolizumab	Paclitaxel and carboplatin	The percentage of patients with a pathological complete response was 64.8% in the pembrolizumab-chemotherapy group and 51.2% in the placebo-chemotherapy group.	(62)
Triple-negative breast cancer	Pembrolizumab	Nab-paclitaxel; paclitaxel; or gemcitabine plus carboplatin	Among patients with a CPS of 10 or more, median PFS was 9.7 months with pembrolizumab-chemotherapy and 5.6 months with placebo-chemotherapy.	(63)
Urothelial cancer	Atezolizumab	Gemcitabine and a platinum	Median PFS in the intention-to-treat population was 8.2 months in atezolizumab plus chemotherapy group and 6.3 months in chemotherapy group.	(64)
Nasopharyngeal carcinoma	Toripalimab	Gemcitabine and cisplatin	A significant improvement in PFS was detected in the toripalimab plus chemotherapy arm compared to the chemotherapy arm (11.7 versus 8.0 months).	(65)

PD-1/L1, programmed death receptor‐1/programmed death protein ligand‐1; SCLC, small cell lung cancer; PFS, progression-free survival; OS, overall survival; CPS, combined positive score.

PD-1/L1 inhibitor has been proven to have synergistic effects with chemotherapy drugs (platinum, paclitaxel, pemetrexed, and etoposide) with a significant improvement in the efficacy in various types of advanced lung cancer (56–59). Based on these and related studies, pembrolizumab in combination with pemetrexed and platinum-based chemotherapy was approved by the FDA in 2018 as the first-line treatment for metastatic non-squamous non-small-cell lung cancer lacking *EGFR* or *ALK* mutations. Nivolumab plus chemotherapy was approved by the FDA for its ability to significantly prolong the OS and progression-free survival (PFS) compared with chemotherapy alone in patients with gastric, gastroesophageal junction, or esophageal adenocarcinoma (60). In esophageal squamous cell carcinoma, combination therapy with pembrolizumab plus 5-fluorouracil and cisplatin was approved by the FDA due to a significantly longer OS and PFS than chemotherapy alone (61). PD-1 inhibitor plus chemotherapy has been approved by the FDA as it provides significant benefits to patients with triple-negative breast cancer compared with chemotherapy alone, both as neoadjuvant therapy and advanced adjuvant therapy (62, 63). Furthermore, PD-1/L1 inhibitor plus chemotherapy significantly extended PFS compared with chemotherapy alone in urothelial and nasopharyngeal cancers (64, 65).

In conclusion, PD-1/L1 inhibitor plus chemotherapy has shown good synergistic efficacy in multiple cancers, which verifies the relevant theoretical research results. At present, many clinical trials are underway in different countries with different PD-1/L1 inhibitors and chemotherapy regimens for different cancers. It is believed that with the completion of these studies, PD-1/L1 inhibitor combined chemotherapy will become the first-line treatment in more and more cancers. However, not all PD-1/L1 inhibitors combined with chemotherapy show synergistic efficacy. In gastric cancer, ovarian cancer, head and neck squamous cell carcinoma, and urothelial carcinoma, some clinical trials have shown no significant advantages of this combined regimen (66–70). The possible reasons are diverse and need further research.

## Research evidence in sarcomas

Compared with studies on the effect of chemotherapy on the TME in other cancers, there are few studies on the effect of chemotherapy on the TME in sarcomas. Nevertheless, current studies have initially elucidated the effects of chemotherapeutic drugs (doxorubicin, cyclophosphamide, gemcitabine docetaxel and trabectedin) that commonly used in sarcomas on TME. Doxorubicin can not only stimulate the anti-tumor immune response by inducing immunogenic cell death (71), it also up-regulated the expression of PD-L1 in both the clinical osteosarcoma tissue samples and the osteosarcoma cell lines (72, 73). Cyclophosphamide can selectively inhibit immunosuppressive cells, including Treg cells and MDSCs, and induce interferon-γ-mediated anti-tumor immune response (74). Gemcitabine can effectively increase the proportion of T cells and the total proportion of infiltrating immune cells, reduce the proportion of MDSCs and macrophages, and promote the expression of PD-L1 on tumor cells (75). Docetaxel-based chemotherapy promotes the intratumor infiltration of T cells and upregulates the abundance of PD-1 and PD-L1 in mouse tumor transplantation model, thereby sensitizing tumor to anti-PD1 blockers (76). Trabectedin selectively reduces monocytes and TAMs in treated tumors and can increase the presence and functional activity of T lymphocytes. In fibrosarcoma models with poor response to PD-1 immunotherapy, treatment with Trabectedin prior to anti-PD-1 can improve antitumor efficacy (77, 78). In addition, an earlier study showed that the chemotherapy drug decitabine facilitates immune recognition of sarcoma cells by upregulating cancer/testis antigens, MHC molecules, and intracellular cell adhesion molecule-1 (79). Another study revealed that chemotherapy could significantly increase the total immune cell infiltration in the TME of sarcoma, especially TAM-2 macrophages, B cells, and CD4+ T cells. Upregulation of genes and cytokines associated with antigen presentation was also observed. An increase in monocyte infiltration is associated with a good pathological reaction (necrosis ≥ 90% after chemotherapy) (80). Additionally, chemotherapy combined with local hyperthermia can lead to increased tumor infiltrating lymphocyte and decreased Treg count in the TME, which is significantly related to a better prognosis (81).

Although there are only a few clinical studies on the efficacy of PD-1/L1 inhibitor plus chemotherapy in sarcomas, some studies have reported improved efficacy (**Table 2**). At present, several retrospective studies have suggested that chemotherapy combined with PD-1 inhibitor has certain efficacy in soft tissue sarcomas (85–87). In these retrospective studies, each sarcoma subtype responded differently to the combination regimen. Overall, undifferentiated pleomorphic sarcomas had the best response. However, the limited nature of retrospective studies limits the level of evidence. In addition to that, several prospective clinical trials have reported promising results. A phase 1/2 non-randomized clinical trial in 2020 showed that pembrolizumab plus doxorubicin was well tolerated. Although the primary end point of ORR was not reached, the PFS and OS compared favorably to those of previous studies (14). The results of another phase 2 clinical trial in 2021 confirmed that the combination of pembrolizumab plus doxorubicin has an efficacy similar to that reported in a previous trial on sarcomas (13). However, some clinical trials did not report synergistic efficacy of PD-1 inhibitor plus chemotherapy in sarcomas. An open label, multi-center phase IB study in 2021 showed that the ORR and PFS in the sarcoma expansion cohort who received durvalumab plus trabectedin treatment were comparable to those in a trabectedin single agent study, and there was no clear evidence of synergistic activity in this unselected population (84). Another clinical trial concluded that pembrolizumab plus cyclophosphamide did not show meaningful activity in sarcoma (82). However, the latest research shows that the efficacy of pembrolizumab plus cyclophosphamide significantly improved in sarcoma patients with tertiary lymphoid structures (83). This topic is worth further research.

**Table 2:** Reports on the efficacy of PD-1/L1 inhibitor plus chemotherapy in sarcomas.

Year of report	Type of study	Type of sarcoma	Therapeutic regimen	Number of cases	Results	References
2018	Phase 2 clinical trial	Sarcoma	Pembrolizumab plus metronomic cyclophosphamide	50	Pembrolizumab plus metronomic cyclophosphamide has limited activity in selected STS.	(82)
2020	Phase 1/2 nonrandomized clinical trial	Anthracycline-naive sarcoma	Pembrolizumab plus doxorubicin	37	Although the primary end-point for ORR was not reached, the PFS and OS compared favorably to those reported in prior studies.	(14)
2021	Phase 2 clinical trial	Soft-tissue sarcoma with tertiary lymphoid structures	Pembrolizumab plus metronomic cyclophosphamide	35	Confirmed that selection based on TLS status is an efficient approach to tailor immunotherapy in STS patients.	(83)
2021	Phase 2 clinical trial	Soft-tissue sarcoma	Pembrolizumab plus doxorubicin	30	Combination pembrolizumab and doxorubicin has manageable toxicity and preliminary promising activity in the treatment of patients with anthracycline-naive advanced STS.	(13)
2021	Phase Ib clinical trial	Soft-tissue sarcoma	Trabectedin plus durvalumab	16	ORR and PFS in the sarcoma expansion cohort were comparable to the ones reported in trabectedin single agent studies, and there was no clear evidence of synergistic activity in this unselected population.	(84)

STS, soft tissue sarcoma; ORR, overall response rate; PFS, progression-free survival; OS, overall survival; TLS, tertiary lymphoid structures.

In conclusion, PD-1 inhibitor plus chemotherapy has shown promising activity in sarcomas, especially in undifferentiated pleomorphic sarcoma, dedifferentiated liposarcoma, and angiosarcoma. However, there is little research in this field, and some studies did not show the synergistic effect of this combination therapy (**Table 2**). The possible reasons are diverse and need further research.

## Ongoing trials on PD-1/L1 inhibitor plus chemotherapy in sarcomas

At present, at least 20 clinical trials of PD-1/L1 inhibitor plus chemotherapy in the treatment of sarcomas have been registered in clinicaltrials.gov and are recruiting (**Table 3**). The PD-1 inhibitors used in these clinical trials include camrelizumab, durvalumab, nivolumab, pembrolizumab, retifanlimab, sintilimab, and Toripalimab. While the chemotherapy drugs are commonly recommended for sarcoma treatment, including doxorubicin, gemcitabine, docetaxel, paclitaxel, ifosfamide, trabectedin, and eribulin. Most of these clinical studies are investigating the treatment of the common sarcoma subtypes, and some of the drugs are also used in the treatment of osteosarcoma and retroperitoneal sarcoma. Several clinical trials are attempting to determine the efficacy of PD-1 inhibitor plus chemotherapy as neoadjuvant therapy for sarcomas.

**Table 3:** Highlighted ongoing clinical trials of PD-1/L1 inhibitor plus chemotherapy for sarcomas.

Indication	Therapy	Setting	NCT ID
Selected retroperitoneal sarcomas	Retifanlimab plus doxorubicin and ifosfamide	Neoadjuvant therapy	NCT04968106
Advanced STS	Camrelizumab plus albumin-bound paclitaxel	Second/more line therapy	NCT05189483
Advanced Sarcoma	Nivolumab plus talimogene laherparepvec and trabectedin	First/more line therapy	NCT03886311
Advanced STS	Durvalumab plus doxorubicin	Second/more line therapy	NCT03802071
Advanced Sarcoma	Metronomic doses of gemcitabine, doxorubicin and docetaxel, and nivolumab	Second/more line therapy	NCT04535713
High-risk STS	Camrelizumab plus liposome doxorubicin and Ifosfamide	Neoadjuvant therapy	NCT04606108
Advanced STS	Anti-PD-1 antibody injection (609A) plus doxorubicin	First/more line therapy	NCT05138146
Selected advanced STS	Sintilimab plus doxorubicin and ifosfamide	First line therapy	NCT04356872
Advanced STS	Doxorubicin plus anti-CTLA-4 antibody AGEN1884 and anti-PD-1 antibody AGEN2034	Second/more line therapy	NCT04028063
Advanced STS	Pembrolizumab plus doxorubicin	First/more line therapy	NCT03056001
Advanced STS	Nivolumab plus trabectedin	Second/more line therapy	NCT03590210
Advanced STS	Nivolumab plus ipilimumab and trabectedin	First line therapy	NCT03138161
Leiomyosarcoma and undifferentiated pleomorphic sarcoma	Pembrolizumab plus gemcitabine	First/more line therapy	NCT03123276
Selected advanced STS	Pembrolizumab pus eribulin	Second/more line therapy	NCT03899805
Advanced STS	Sintilimab plus adriamycin and ifosfamide	First/more line therapy	NCT04589754
Advanced bone and soft tissue sarcoma	Toripalimab plus CAV/IE	Second/more line therapy	NCT04589741
Selected advanced STS	Camrelizumab plus doxorubicin	First line therapy	NCT04910126
Advanced STS	Durvalumab plus trabectedin	Second/more line therapy	NCT03085225
Non-metastatic and locally resectable osteosarcoma	Camrelizumab plus adriamycin, cisplatin, ifosfamide and methotrexate	Neoadjuvant therapy	NCT04294511
High-grade osteosarcoma	Camrelizumab plus MAPI	Second line therapy	NCT04351308

NCT ID, Registration number on https://clinicaltrials.gov; STS, soft tissue sarcoma; CAV/IE, cyclophosphamide, adriamycin, vincristine, Ifosfamide and Etoposide chemotherapy; MAPI, methotrexate, adriamycin, cisplatin and ifosfamide chemotherapy.

The completion of these clinical trials will further validate the synergistic efficacy of different PD-1/L1 inhibitors plus chemotherapeutic agents in the treatment of sarcoma. This is a necessary step to improve the therapeutic outcome of sarcoma patients. However, these clinical trials are only conducting preliminary verification, and some deep-seated and detailed problems have not been examined. The major problems with these trials include 1) not selecting a specific sarcoma subtype; 2) Many treatment regimens are not based on solid theoretical and preclinical trials; 3) The combination of chemotherapy drugs and immunotherapy drugs is too mechanical, and the influence of dose and timing of administration is not considered; 4) The effect of adjuvant medication was not considered.

## Discussion

We first reviewed the synergistic mechanism of PD-1/L1 inhibitor plus chemotherapy in the treatment of cancers and the effectiveness of this combined regimen in many cancers and then summarized the current evidence on the application of this combined regimen in the treatment of sarcomas as well as the main ongoing clinical trials. The clinical efficacy of this combination therapy in lung cancer, gastroesophageal cancer, and breast cancer has been proven, and it has been approved by the FDA. PD-1/L1 inhibitor plus chemotherapy is an important breakthrough in the treatment of many cancers, which requires further research for more progress.

**Figure 2:**
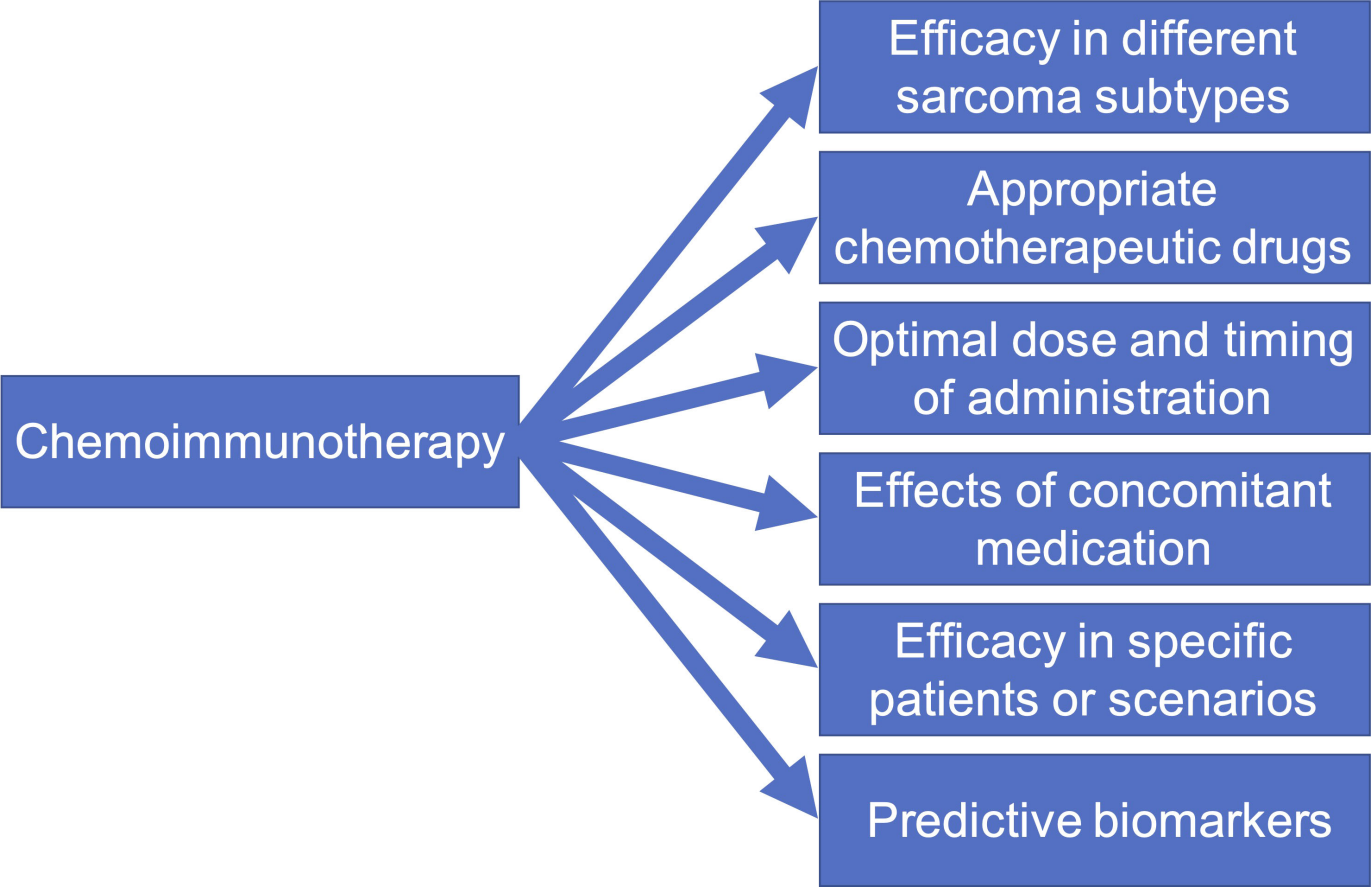
Six future directions for mechanism and clinical studies on PD-1/L1 inhibitor plus chemotherapy in soft-tissue sarcomas.

However, the application of PD-1/L1 inhibitor plus chemotherapy in sarcoma is still in the preliminary verification stage. To improve the therapeutic outcome in sarcomas, the research on PD-1/L1 inhibitor plus chemotherapy in sarcomas needs to be more in-depth and detailed (**Figure 2**). There are various subtypes of sarcomas, and the drug sensitivity of different sarcomas varies greatly (88). Single-agent immunotherapy may be appropriate in some sarcoma subtypes. The first step should be to determine which sarcoma subtypes are more likely to respond to treatment with combination regimens, and validate each sarcoma subtype individually (89). Second, chemotherapeutic drugs suitable for combination with PD-1/L1 inhibitor should be screened. Obviously, the interaction mechanism and effect of each chemotherapeutic drug with the PD1/L1 inhibitor may be different. Some drugs are suitable for combination with PD1/L1 inhibitor, while others are not. For example, it has been demonstrated that the use of glucocorticoids while immunotherapy will lead to poor prognosis (90). Therefore, chemotherapy drugs that need to be accompanied by glucocorticoids may not be suitable for combination with immunotherapy. In addition, different sarcoma subtypes have different sensitivities to different chemotherapy drugs. Therefore, it is necessary to choose different combination chemotherapy agents for different sarcoma subtypes. Another important problem neglected at present is the dose of chemotherapeutic drugs and the timing of administration of PD-1/L1 inhibitor, which can have a great impact on the therapeutic effectiveness (34, 91). Excessive doses of chemotherapeutic drugs inevitably lead to severe immunosuppression and weaken the efficacy of PD-1/L1 inhibitor. If the timing of administration is not appropriate, T cells activated by PD-1/L1 inhibitor will also be reduced by chemotherapy and inhibit the efficacy of anti-tumor immunity. The effects of concomitant medication have also not been considered. There are many concomitant drugs in chemotherapy, many of which have been preliminarily confirmed to inhibit the efficacy of PD-1/L1 inhibitor (92). Therefore, the concomitant medication should be considered in detail in the design of various clinical trials. The safety and efficacy of combination therapy in different application scenarios are also worth exploring, such as in elderly or juvenile patients, or under the setting of neoadjuvant therapy. Finally, efficacy markers require further investigations. At present, high PD-L1 expression, microsatellite instable-high (MSI-H)/mismatch repair deficient (dMMR) phenotype, and tumor mutation burden-high status (TMB-H) have been proposed as a predictive biomarker to predict response to PD-1 inhibitors in many cancers (93). However, sarcomas with one of these three characteristics are rare, and the available evidence has not found that they can predict the response of sarcomas to PD-1 inhibitors (94, 95). Further clinical trials are needed to confirm whether chemotherapy can change the sensitivity of sarcomas with high PD-L1 expression, MSI-H/dMMR phenotype, or TMB-H to immunotherapy.

Although PD-1/L1 inhibitor plus chemotherapy has demonstrated synergistic efficacy in some cancers, there are some problems that cannot be ignored, including chemotherapy toxicity, immunosuppressive effect of chemotherapeutic or concomitant drugs, and ineffective clinical trial results. Obviously, these problems need to be explored by future clinical studies. Despite these doubts, it cannot be denied that PD-1/L1 inhibitor plus chemotherapy is a promising treatment for sarcomas. We believe that this combined approach will play an important role in the treatment of some subtypes of sarcoma in the future.

## Author contributions

All authors listed have made a substantial, direct, and intellectual contribution to the work, and approved it for publication.

## Conflict of interest

The authors declare that the research was conducted in the absence of any commercial or financial relationships that could be construed as a potential conflict of interest.

## Publisher’s note

All claims expressed in this article are solely those of the authors and do not necessarily represent those of their affiliated organizations, or those of the publisher, the editors and the reviewers. Any product that may be evaluated in this article, or claim that may be made by its manufacturer, is not guaranteed or endorsed by the publisher.
